# Evolution of Airway Inflammation in Preschoolers with Asthma—Results of a Two-Year Longitudinal Study

**DOI:** 10.3390/jcm9010187

**Published:** 2020-01-09

**Authors:** Paraskevi Xepapadaki, Paraskevi Korovessi, Claus Bachert, Susetta Finotto, Tuomas Jartti, John Lakoumentas, Marek L. Kowalski, Anna Lewandowska-Polak, Heikki Lukkarinen, Nan Zhang, Theodor Zimmermann, Nikolaos G. Papadopoulos

**Affiliations:** 1 Allergy Department, 2nd Pediatric Clinic, National and Kapodistrian University of Athens, 11527 Athens, Greece; vkorovessi@gmail.com (P.K.); john.lakoo@gmail.com (J.L.); ngpallergy@gmail.com (N.G.P.); 2 Upper Airways Research Laboratory, Ghent University Hospital, 9000 Ghent, Belgium; Claus.Bachert@ugent.be (C.B.); Nan.Zhang@UGent.be (N.Z.); 3 Department of Molecular Pneumology, Friedrich-Alexander-Universität Erlangen-Nürnberg, Universitätsklinikum Erlangen, 91054 Erlangen, Germany; Susetta.Neurath-Finotto@uk-erlangen.de; 4 Department of Paediatrics and Adolescent Medicine, Turku University Hospital and University of Turku, 20520 Turku, Finland; hepelu@utu.fi (H.L.); tuomas.jartti@utu.fi (T.J.); 5 Department of Immunology, Rheumatology and Allergy, Medical University of Lodz, 92213 Lodz, Poland; marek.kowalski@umed.lodz.pl; 6 Department of Rheumatology, Medical University of Lodz, 92213 Lodz, Poland; anna.lewandowska-polak@umed.lodz.pl; 7 Department of Pediatrics and Adolescent Medicine, Dept of Allergy and Pneumology, Children’s Hospital, Friedrich-Alexander-Universität Erlangen-Nürnberg, Universitätsklinikum Erlangen, 91054 Erlangen, Germany; Theodor.Zimmermann@uk-erlangen.de; 8 Division of Infection, Immunity & Respiratory Medicine, University of Manchester, Manchester M13 9PL, UK

**Keywords:** asthma, PreDicta, preschool, FeNO, spirometry

## Abstract

Fractional exhaled nitric oxide (FeNO) is a non-invasive marker for eosinophilic airway inflammation and has been used for monitoring asthma. Here, we assess the characteristics of FeNO from preschool to school age, in parallel with asthma activity. A total of 167 asthmatic children and 66 healthy, age-matched controls were included in the 2-year prospective PreDicta study evaluating wheeze/asthma persistence in preschool-aged children. Information on asthma/rhinitis activity, infections and atopy was recorded at baseline. Follow-up visits were performed at 6-month intervals, as well as upon exacerbation/cold and 4–6 weeks later in the asthmatic group. We obtained 539 FeNO measurements from asthmatics and 42 from controls. At baseline, FeNO values did not differ between the two groups (median: 3.0 ppb vs. 2.0 ppb, respectively). FeNO values at 6, 12, 18 and 24 months (4.0, CI: 0.0–8.6; 6.0, CI: 2.8–12.0; 8.0, CI: 4.0–14.0; 8.5, CI: 4.4–14.5 ppb, respectively) increased with age (correlation *p* ≤ 0.001) and atopy (*p* = 0.03). FeNO was non-significantly increased from baseline to the symptomatic visit, while it decreased after convalescence (*p* = 0.007). Markers of disease activity, such as wheezing episodes and days with asthma were associated with increased FeNO values during the study (*p* < 0.05 for all). Age, atopy and disease activity were found to be important FeNO determinants in preschool children. Longitudinal and individualized FeNO assessment may be valuable in monitoring asthmatic children with recurrent wheezing or mild asthma.

## 1. Introduction

The use of objective measurements, including the measurement of lung function and airway inflammation by means of fractional exhaled nitric oxide (FeNO) is currently becoming reinforced in most official recommendations for children with asthma, even though there are not always clear correlations between the two. Moreover, associations between disease activity and airway inflammation are frequently inconclusive or negative, probably because they have mostly been assessed cross-sectionally or for limited time periods [1,2].

As defined by the Global Initiative for Asthma (GINA), FeNO measurements are associated with clinical asthma control indices in school-aged children and adults [3]. However, the preschool-age group is of particular interest because preschool age is often a milestone whereby wheezing disease is expected to transform by either resolving or becoming persistent in a significant number of patients. Few studies, however, have evaluated how inflammation progresses or correlates with symptoms in asthmatic preschoolers [4,5,6]. A limited number of cross-sectional studies have shown that preschool children with increased wheezing morbidity, as assessed by symptom frequency and persistence, present higher levels of FeNO, which suggests that FeNO levels might correlate with current and/or subsequent asthma diagnosis [7,8]. Moreover, fluctuations in airway inflammation have not been extensively studied in young children with asthma-associated symptoms, either due to difficulties in obtaining acceptable and repeatable maneuvers or to the potential impact of viral respiratory infections, use of inhaled corticosteroids and atopy per se [9].

In order to understand wheeze/asthma in preschoolers and how it relates to asthma later in life, we need to understand the main characteristics of obstructive airway disease over time, particularly bronchoconstriction, inflammation and hyper-responsiveness. The PreDicta pediatric longitudinal cohort, within “PreDicta”, a European Commission-funded project under the 7th Framework Program for Research and Technological Development (FP7), was designed as a 2-year prospective study with the aim of evaluating wheeze/asthma persistence in preschool- to school-age children [10]. Herein, we evaluate the evolution of airway inflammation from preschool to school age, in parallel with disease activity.

## 2. Methods

### 2.1. Study Population

The PreDicta cohort study was conducted across five major cultural and climatic regions of Europe: Greece, Germany, Belgium, Poland and Finland. Children 4–6 years of age with an asthma diagnosis of mild to moderate severity according to GINA [11] confirmed by a doctor from one of the participating study centers within the last 2 years were invited to participate as cases. Moreover, healthy, age-matched children with no reported history of wheeze/asthma served as cross-sectional controls at the beginning of the 2-year follow-up period. The eligibility and exclusion criteria, study design and baseline characteristics of the cohort have been described previously [10]. The study was approved by all participants’ institutional ethics committees and written informed consent was obtained from parents.

### 2.2. Study Design

At baseline, children were required to be asymptomatic, without a cold or an exacerbation for at least 4 weeks. A questionnaire including detailed information on asthma and rhinitis activity and severity and infection history was filled in by all participants, while the presence of atopy was assessed by skin prick tests (SPTs) to common aeroallergens [10].

Follow-up visits were performed at 6-month intervals for two years in the asthmatic group. At regular follow-up visits, a questionnaire with information regarding asthma and rhinitis activity and infections during the previous period was obtained. Moreover, children were assessed in the clinic during symptomatic periods on the basis of their daily symptom score or their parent’s perception and were reassessed 4–6 weeks later at convalescence, through a questionnaire and clinical examination. At all of the time points, airway inflammation was determined with FeNO measurements in parts per billion (ppb), using the Bedfont NObreath equipment (Bedfont Ltd., UK) [12]. Data were available from the four follow-up visits for 146, 125, 121 and 140 subjects, respectively. Measurements were also performed during 75 symptomatic visits and 56 of the respective convalescent visits.

### 2.3. FeNO Measurements

FeNO values were measured at visits using the Bedfont NObreath equipment (Bedfont Ltd., Maidstone, UK), in parts per billion (ppb). According to the 2005 American Thoracic and European Respiratory Society’s guidelines [12] and the Bedfont NObreath specifications, for a correct FeNO measurement, a single breath sample was instantly analyzed after the subject inhaled to total lung capacity through a NO-scrubbing filter to avoid contamination with ambient NO. All exhalations were performed with an exhalation pressure of 10 to 20 cm H_2_O to maintain a fixed flow rate of 50 ± 5 mL/s (<6 attempts per visit). The pressure is necessary to ensure closure of the soft palate, to avoid contamination of the FeNO with gas from the nasopharynx where NO concentrations are very high. In addition, the targeted constant-flow exhalation rate consists of a washout phase followed by a 2-s NO plateau. It is generally considered that this plateau represents NO derived primarily from the lower respiratory tract. A ball moving within frames was used to help young children blow at the right pressure during the exhalation. Whenever possible, the two most consistent attempts (i.e., lasting 6–10 s and/or agree within 10%) were recorded. Children had not eaten or drunk anything for at least 2 h before and avoided nitrate-rich meals for at least 20 h before the measurements. Directions were given according to these dietary recommendations for regular visits. Exacerbation visits were arranged according to the last meal or snack. FeNO analysis was performed before spirometry because spirometric maneuvers have been shown to transiently reduce airway inflammation measurements.

The following algorithm was used in order to calculate FeNO values at different time points: (a) if at least one value was 0 or 1 ppb, the maximum value was recorded; (b) if both values were greater than 1, then (b._1_) if the absolute difference was ≤10 ppb, we calculated the average value, or (b._2_) in case the absolute difference was >10 ppb, the maximum value was recorded. Personal best FeNO was defined as the minimum value recorded in all visits, baseline and follow-up for all cases.

### 2.4. Predictors of FeNO Measurements

Disease activity markers were derived from the questionnaires obtained at baseline and during symptomatic/convalescent periods. These were selected from the following guidelines (a) the GINA guidelines for determining asthma control and severity level, on the basis of criteria such as frequency of day and night asthma symptoms, night-time awakenings due to asthma, limitation of activities, need for reliever and controller medication and hospitalizations for asthma; (b) the PRACTALL consensus for asthma phenotyping [13]; and (c) the Allergic Rhinitis and its Impact on Asthma (ARIA) initiative for assessing the frequency and severity of rhinitis symptoms [14]. Moreover, history of infections including the frequency and duration of upper and lower respiratory infections, the use of antibiotics and family history of atopy-associated diseases were included as predictors. Symptoms recorded in diary cards during symptomatic and convalescent visits were used for comparisons. All the predictors that were assessed are depicted in the Supplementary Materials, Table S1.

### 2.5. Statistical Analysis

All variables assessed were non-parametric, according to the Shapiro-Wilk test for composite normality; thus, descriptive statistics are presented as medians (25–75 percentiles—IQRs). In order to identify dependencies between FeNO and other variables, non-parametric tests were applied: the Wilcoxon rank-sum test was used for qualitative variables and Kendall’s correlation was used for quantitative variables (such as the subjects’ age).

The Friedman test was used in order to compare values at three consecutive visits (baseline or follow-up, symptomatic and convalescent) for each patient. Post-hoc analysis was carried out using the Wilcoxon paired signed-rank test, along with the Bonferroni correction.

All tests were considered two-sided and statistical significance was defined as *p* < 0.05. Statistical analysis was performed with the R software for statistical computing, along with the RStudio interface (both open-source products).

## 3. Results

A total of 167 cases (102 males, 61%, mean age: 5.2 ± 0.7) and 66 controls (30 males, 45%, mean age: 5.1 ± 0.8) were recruited from the five participating centers. The demographic characteristics of the two groups at inclusion have been reported elsewhere [10]. FeNO measurements were available for 131 (79.6%) asthmatic children (median: 3.0, CI: 0.0–6.7 ppb) and 35 (53%) healthy controls (median: 2.0, CI: 0.0–7.2 ppb, *p* = 0.37).

Baseline characteristics regarding disease activity and atopic history of the asthmatic children are depicted in Table 1.

Atopic asthmatics presented increased FeNO levels compared to non-atopic asthmatics, however, the results were not statistically significant either when assessed in terms of the occurrence of any positive SPT (median: 3.0, CI: 0.0–6.9 vs. 2.0, CI: 0.0–5.2, respectively, *p* = 0.47) or by quantifying, i.e., by summation of positive SPTs (descriptive: 4.8, *p* = 0.48). Reported personal and family history of atopy-related diseases was not associated with increased FeNO levels at baseline. 

### 3.1. FeNO Evolution during the Two-Year Follow-Up

During the visits we obtained a total of 42 FeNO measurements from controls (35 at baseline) and 539 from cases. FeNO was measured in 67.8% of children at 6 months of follow-up (median: 4, CI: 0.0–8.6 ppb), 74.4% at 12 months (median: 6.0, CI: 2.8–12.0 ppb), 83.5% at month 18 (median: 8.0, CI: 4.0–14.0 ppb) and 72.1% at the final visit (median: 8.5, CI: 4.4–14.5 ppb), suggesting an increasing trend with time/age in asthmatics.

**Figure 1:**
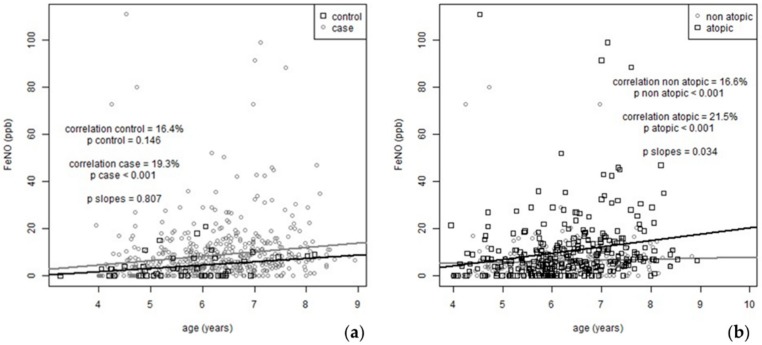
(**a**) Non-parametric correlation between subjects’ fractional exhaled nitric oxide (FeNO) and age, separately assessed in controls and cases. “P slopes” denote the p-value of the comparison between the slopes of the two separate trend lines, i.e., controls (black line) vs. cases (gray line). (**b**) FeNO progression slopes in preschool asthmatics for atopics (black line) and non-atopics (gray line), at baseline and during the 2-year follow-up.

Kendall’s correlation reveals a correlation of 16.4% between FeNO value and age in controls (*p* = 0.14) and a correlation of 19.3% (*p* < 0.001) in children with asthma (Figure 1a). Post-hoc power analysis yielded powers of 18% and 99%, respectively, for the above outcomes at the significance level of 0.05; however, the slopes of the corresponding linear fits do not differ significantly between cases and controls (*p* = 0.8). With regards to atopy, children with at least one positive SPT presented a significantly increased FeNO trajectory compared to non-atopics (*p* = 0.03, Figure 1b).

At all time points, no differences were noted in FeNO values in respect to sex when assessed cross-sectionally in asthmatics.

### 3.2. FeNO and Seasonality

The FeNO values, either as absolute or % change from baseline values, were independent of the season when they were assessed, at all visits; nevertheless, the number of symptomatic visits (either due to a cold or an exacerbation) significantly increased during “cold months” (autumn = 26, winter = 27, spring = 16, summer = 8).

### 3.3. Variation in FeNO Values from Healthy State (Asymptomatic) to Exacerbation and Convalescent Visit

**Figure 2:**
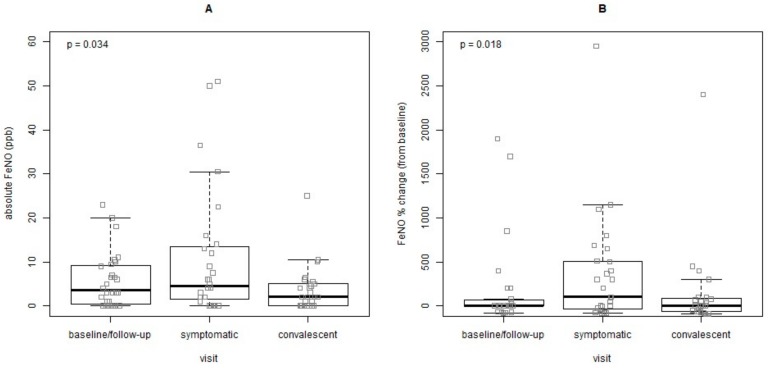
Variations in FeNO measurements from healthy state (baseline/follow-up) to the subsequent exacerbation and convalescence periods in preschool-aged children with recurrent wheeze/asthma. (**A**) Comparison of absolute FeNO values, (**B**) Comparison of FeNO % change.

**Figure 3:**
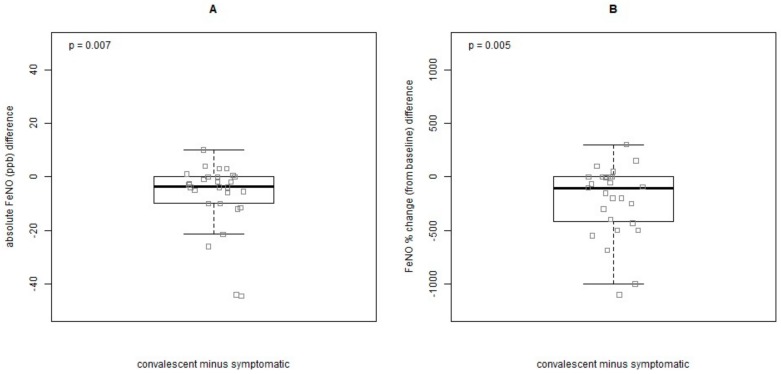
Difference in FeNO values of preschoolers with asthma during exacerbations and at their respective convalescent visits (**A**) in absolute values, (**B**) in FeNO % change (from baseline).

In order to evaluate the variation in FeNO levels in asthmatic children during periods of different health status, a comparison was performed at three distinct time points: (a) either at baseline or during the last follow-up visit before a symptomatic visit, when the child was still asymptomatic; (b) at the subsequent symptomatic visit; and (c) at the respective convalescent visit. Forty-nine children (65.3%) were able to perform FeNO measurements upon exacerbation and forty-two (75%) at convalescence. In total, 28 measurements were available for comparison for all three time points. A post-hoc power analysis yielded a power of 66% for the following outcomes at the significance level of 0.05. There was an increase in absolute FeNO values from the baseline to the symptomatic visit, although there was no significant difference (baseline median: 3.5, CI: 0.7–9.1 ppb vs. symptomatic median: 4.5, CI: 1.7–13.2, respectively, *p* = 0.32).A significant drop was observed between the symptomatic and the convalescent visit (median: 2.0, CI: 0.0–5.0, *p* = 0.007), (Figure 2A). The % FeNO differences were also significant from symptomatic and convalescent visits (symptomatic visits: median 100, CI: −38.6–504.1, convalescent: median 0.0, CI: −62.8–87.5, *p* = 0.005) (Figure 2B). The differences from symptomatic to convalescent visits in absolute (*p* = 0.007) and % FeNO values (*p* = 0.005) are depicted in Figure 3A,B. The effect of inhaled corticosteroids (ICS) could not be assessed since most asthmatic children were on prophylactic treatment at all three time points (baseline: 80.7, symptomatic: 76.7, convalescent: 81.6).

### 3.4. Predictors of FeNO Measurements at Baseline and during the Two-Year Follow-Up

Predictors derived from the questionnaires obtained at all-time points were assessed in respect to FeNO values (Supplementary Materials, Table S1). FeNO measurements at baseline were significantly correlated with the number of reported asthma episodes in the preceding 12 months (correlation coefficient: 13.7, *p* = 0.03), while wheezing episodes and the number of days with asthma symptoms were positively associated with increased FeNO values at the end of the 2-year follow-up (median: 19.5, IQR: 15.75–46.25 and correlation coefficient: 16.1, *p* = 0.04, respectively).

## 4. Discussion

In our cohort, FeNO levels did not differ significantly between “asthmatic”/recurrent wheezers when asymptomatic (at baseline) and healthy preschool children. A gradual and significant increase in airway inflammation was noted in asthmatics in respect to time/age when prospectively assessed; however, slopes between asthmatics and controls did not differ. In the presence of atopy, as assessed by SPTs, asthmatics presented significant increases in airway inflammation during the study. Moreover, FeNO values were significantly associated with markers of asthma activity such as the number of days with asthma symptoms reported in the preceding 12 months and the number of asthma exacerbations during the 2-year follow-up period.

FeNO levels, in both asthmatic and healthy children, were within normal limits, as indicated by international guidelines [12] and in line with previous studies, suggesting that FeNO values in preschoolers are less than 10 ppb [15]. Increased FeNO values have been reported previously in preschoolers with recurrent or persistent asthma-associated symptoms compared to healthy controls [16]; however, this was not the case in our cohort, which is probably due to mild to moderate disease severity [10] or to the fact that measurements were performed during asymptomatic periods at baseline. The lack of difference between the groups with regard to the increase in the FeNO slopes in the 2-year follow-up might suggest that, potentially, FeNO increases are age-dependent, as noted in previous studies [15], and independent of disease activity in this age group, although the impact of small size on controls cannot be excluded. Nevertheless, atopy per se was a significant risk factor for the increased FeNO trajectory in asthmatics, which is in line with reports supporting a strong association between FeNO and allergic sensitization [17].

During episodic periods, FeNO levels significantly increased, while 4–6 weeks later, the values recorded were even lower than their respective “baseline” ones. High levels of FeNO are typically considered a marker of airway eosinophilic inflammation and are associated with deterioration in asthma control [18]. FeNO levels increase during virus-induced asthma exacerbations, which are mainly attributed to rhinoviruses, and reflect epithelial host-defense responses [19]. In our cohort, viral respiratory infections, mostly by rhinoviruses, were the most commonly identified triggers for asthma worsening (manuscript in preparation). It is plausible that the intermittent inflammatory airway response that follows an episode accounts for the brief increase in FeNO levels. It is well-established that FeNO levels are affected by the intake of corticosteroids, either oral or inhaled, to the extent that the use of FeNO has been proposed as a marker for glucocorticoid response, or alternatively for adjusting ICS dosing. The vast majority of asthmatic preschoolers in our cohort (95%) were on prophylactic treatment at baseline, as described elsewhere [10]; thus, this may account for the low FeNO values noted at inclusion. Moreover, treatment with increased doses of inhaled corticosteroids, which is frequently recommended as an add-on therapy to b2-agonists during exacerbations [20], may explain the lower FeNO levels noted at convalescence [21]. This further suggests that treatment may improve airway inflammation, even if children appear asymptomatic.

We recorded FeNO values in 75.5% of all scheduled visits on average, and in 65% of visits during episodes in asthmatics, indicating that FeNO determination is feasible in most, but not all preschoolers, following proper training. Reliable FeNO measurements can be obtained in a standardized way from the age of 4 years. Reports have shown that the NObreath equipment provides results that are comparable with other techniques [22]. In addition, FeNO measurements were performed during morning hours, thus excluding the possibility of bias due to circadian fluctuations [23].

Our data support the monitoring of asthma with objective measurements, even in preschoolers. FeNO personalized monitoring (±bronchodilator reversibility) was reported to be dominate over other diagnostic tests, while an economic analysis indicated that FeNO monitoring could have value as such a strategy [24]; thus, most recent guidelines suggest the incorporation of FeNO into the asthma management algorithm [25]. Recently, an increasing number of reports have emerged that utilize the measurement of FeNO at multiple expiratory flows and mathematical modeling of pulmonary NO dynamics in order to estimate different components of FeNO, i.e., alveolar or acinar; nevertheless, only a few publications have reported values in children with asthma and no data are available for preschoolers. [26,27,28]. Future research is needed in order to establish the usefulness of the aforementioned FeNO technique in clinical practice, especially in the pediatric population.

All in all, we were able to demonstrate significant fluctuations in airway inflammation, depending on the activity and severity of the disease, which indicates that an individualized approach, i.e., using the personal best as a reference value, may be preferable for this age group. Nevertheless, the age- and atopy-dependent increase needs to be taken into account, suggesting that regular baseline measurement may be necessary. FeNO changes, at least in preschoolers, should be longitudinally and individually assessed, in order to efficiently aid disease management.

The major strength of our study is its multinational character and longitudinal design. Diagnosis and disease assessment were made by specialists, while airway inflammation and atopy were quantified via objective standardized measures. We have previously shown that our cohort is powered to prospectively evaluate the role of infection on asthma persistence and it is representative of preschool asthma, with well-balanced demographic and atopy-related characteristics [10]. Children were assessed for an extended time period at regular follow-up visits and during symptomatic and convalescence periods. We obtained a significant number of FeNO measurements at baseline and regular follow-up visits (561) in the cohort. Although the available number of measurements for comparison during symptomatic periods and convalescence was small, the statistical power obtained was moderate, due to the moderate effect size (significant differences in the compared distributions’ median values) that was present.

However, a weakness of this study is that several parameters, including asthma activity, severity and medication use were assessed based on parental reports, which are subject to recall bias, although previous studies have proven that short-term parental reports can be accurate.

## 5. Conclusions

This study clearly shows that indices of asthma and rhinitis activity and the presence of atopy in preschool-aged children with asthma are significantly correlated with airway inflammation as assessed by longitudinal follow-up. Airway inflammation increases during acute events, then values return to better than the “personal normal” values 4–6 weeks later, suggesting a treatment effect. Thus, longitudinal assessment of FeNO measurements can be valuable for monitoring preschool children with recurrent wheeze/asthma. However, estimations can only be based on personalized, rather than reference values.

## Figures and Tables

**Table 1:** Baseline characteristics regarding disease activity and atopic history of the asthmatic children.

Atopic History Disease Activity	Baseline Characteristics	*n*
Atopic history	Family history of atopic disease (yes), *n* (%)	94 (91)
	Family history of asthma/rhinitis (yes), *n* (%)	89 (86)
	Any positive skin prick test (yes), *n* (%)	70 (56)
Upper Respiratory Symptoms	Rhinitis symptoms are present	
	<4 days/week, *n* (%)	61 (47)
	Rhinitis duration	
	<4 consecutive weeks, *n* (%)	67 (51)
	Are rhinitis symptoms associated with…?	
	Sleep disturbance (yes), *n* (%)	65 (89)
	School impairment (yes), *n* (%)	17 (24)
	Leisure—sport (yes), *n* (%)	35 (49)
	Visual analogue scale, median (CIs)	5 (3–6)
	Number of medication courses for rhinitis in the last 12 months, median (CIs)	1 (0–5)
Lower Respiratory Symptoms	Days with symptoms in the last 3 months	
	<1/week, *n* (%)	83 (64)
	>1/week but <1/day, *n* (%)	37 (28)
	daily, *n* (%)	11 (8)
	Nights with symptoms in the last 3 months	
	≤2 times/month, *n* (%)	81 (62)
	>2 times/month, *n* (%)	15 (12)
	>1/week, *n* (%)	24 (18)
	daily, *n* (%)	11 (8)
	Cough, wheeze or difficulty in breathing during or after exercise in the last 12 months (yes), *n* (%)	84 (64)
	Limitation of activities limited by asthma symptoms (yes), *n* (%)	46 (35)
	Child completely well between symptomatic periods (yes), *n* (%)	92 (70)
	Number of episodes of wheezing/asthma/cough in the last 3 months, median (25–75 percentiles)	1 (1–2)
	Number of episodes of wheezing/asthma/cough in the last 12 months, median (CIs)	5 (3–8)
	Inhaled corticosteroids as prophylactic treatment (yes), *n* (%)	107 (82%)

## Supplementary Materials

The following are available online at https://www.mdpi.com/2077-0383/9/1/187/s1, Table S1: Disease activity predictors during baseline and 2-year follow-up.
