# Supplementary material for: Evolution of Airway Inflammation in Preschoolers with Asthma—Results of a Two-Year Longitudinal Study
Source: J Clin Med. 2020 Jan 9;9(1):187. doi: 10.3390/jcm9010187 (PMC7020050; doi:10.3390/jcm9010187)
Supplement: Supplementary file 1 [file jcm-09-00187-s001.pdf]

Journal of Clinical Medicine

MDPI

Table S1. Disease activity predictors during baseline and 2-year follow-up.

	Baseline	Follow-up	Symptomatic Visit
Upper respiratory symptoms	Rhinitis symptoms are present: <4days/week, >4 days/week	Rhinitis symptoms last 6 months (yes)	Runny nose
	Rhinitis duration<br><4 consecutive weeks, >4 consecutive weeks	Rhinitis symptoms are present: <4days/week, >4 days/week	Stuffy nose,
	Are rhinitis Symptoms associated with?<br>sleep disturbance (no / yes)<br>school impairment (no / yes)<br>leisure/sport (no/yes)	Rhinitis duration:<br><4 consecutive weeks, >4 consecutive weeks	Sneezing
	Visual analogue scale	Are rhinitis symptoms associated with?<br>sleep disturbance (no / yes)<br>school impairment (no / yes)<br>leisure/sport (no/yes)	
	Number of medication courses for rhinitis last 12 months	Visual analogue scale	
		Number of medication courses for rhinitis last 12 months	
Lower respiratory symptoms	Days with symptoms last 3 months:<br>< 1/week, >1week but <1/day, daily	Asthma episodes since last evaluation	Cough during the day/night
	Nights with symptoms last 3 months: ≤2 times/month, >2 times/month, >1/week, almost daily	How many days has the child had coughing, wheezing, shortness of breath, or chest tightness	Wheezing during the day/night
	Cough, wheeze or difficulty in breathing during or after exercise last 12 months (no/yes)	How many days has the child had awakened at night because of symptoms	Difficulty in breathing during the day/night
	Limitation of activities limited by asthma symptoms (no/yes)	How many days has the child had awakened in the morning with symptoms that did not improve within 15min of using a short-acting inhaled b2 agonist	Shortness of breath

Child completely well between symptomatic periods (no/yes)	How many days has the child had symptoms while exercising or playing	Respiratory rate
Number of episodes of wheezing/asthma/cough in the last 3 months	How many days has your child's asthma caused him her to miss school	Wheeze
Number of episodes of wheezing/asthma/cough in the last 12 months	How many days has your child' s asthma caused him her to reduce his/ her activities	Existence/severity of exacerbation: None, mild, moderate, severe
Number of hospitalizations for wheezing/asthma/cough in the last 12 months	Has the child had any emergency visit for its asthma	
Inhaled corticosteroids as prophylactic treatment (no/yes/quantity)	Has the child been hospitalized for its asthma	
	Reliever asthma medication	
	Daily asthma controller medication	
